# Have We a Code of Ethics?

**Published:** 1891-03

**Authors:** 


### Editorial.

### HAVE WE A CODE OF ETHICS?

The constant allusion to a code of ethical laws governing the dental profession has for many years led to the inquiry, Is this supposed code a written set of rules, authorized by and prepared under the supervision of a recognized representative body, or is it simply a collection of moral statutes that should govern individual and all large bodies engaged in a common occupation? The search for an authority in the matter has failed to show any such authorized code. The only one extant at the present time, as far as known, is that adopted by the American Dental Association, August, 1866, which was, doubtless, copied and modified to suit conditions from that of the American Medical Association. This, while excellent in itself, has no binding power, as the authority from which it emanated was not then, nor has it ever been a truly representative organization, and hence could not speak authoritatively on this important matter. Local societies have in various places adopted rules for the government of their members which, while valuable in a restrictive sense, possess no general influence on the profession.

Rules are but the expression of mental growth, and, whether applied to individuals or societies, exhibit only the progress made in moral elevation, and while suitable for one period may not meet all the needs of an ever-growing present or the ethical extensions required for the future.

It may be said, Why formulate rules at all? Why not leave this to the ever-indwelling consciousness of right and wrong in the individual? The answer must be found in the fact that agreement upon such a basis is impossible, and in the end confusion will be the legitimate result. While laws are always unsatisfactory and an abridgment of personal liberty, it seems necessary to have them for good government and for the orderly arrangement of many minds into something akin to unity. If this be true, it seems that the time is ripe for a readjustment of this so-called code. If the American Dental Association changes its character to a truly representative body, a change possible and probable in the near future, one of its first duties should be to arrange the ethical status of the profession in the United States.

The thought that led to this has been the constant violation of what may be regarded as duties each and all owe to every member of a common calling. It has been apparent in many ways and has led to bickering and estrangement in certain circles where only harmony should exist. It is unnecessary to point out in detail these sins of commission and, we may say, omission, for oftentimes an individual is injured as much by silence as by more pronounced speech. This fact must be apparent to all readers of dental journals. It is not unusual to find papers received with favor in societies where all the ideas and, oftentimes, modes of treatment are bodily incorporated, and no attempt made at reference to the originator. The wrong of such a procedure is obvious and works injury not only to those who have laboriously performed original work, but to the person adopting it, for such laxity of morals is always double-acting in its effects, and ends in the debasement of the individual. Much of this trouble might be avoided by a close following of the golden rule; but this, unfortunately, is rarely done.

We have had in many places proper discussions on patents and the rights of dentists under the patent laws and the relation of such to the code; but little or nothing is heard of that deeper and more dangerous enemy to our peace, insidious attacks by anonymous writers intended to undermine influence, and which frequently accomplish not only this but the destruction of hardly-earned reputations. It is time that the thinly-veiled venom displayed in letters to the dental periodicals should be discountenanced by editors and readers.

In the absence of a code of authority, can we not substitute the higher law to deal justly by all men and meet them upon all occasions with a broad and generous spirit, and with an earnest effort to arrive at the truth without fear of an open assault, on the one hand, or of a craven stabbing in the dark, on the other?

